# Systematic evaluation of urinary formic acid as a new potential biomarker for Alzheimer’s disease

**DOI:** 10.3389/fnagi.2022.1046066

**Published:** 2022-11-30

**Authors:** Yifan Wang, Ying Wang, Jinhang Zhu, Yihui Guan, Fang Xie, Xiao Cai, Jiale Deng, Yan Wei, Rongqiao He, Zhuo Fang, Qihao Guo

**Affiliations:** ^1^Department of Gerontology, Shanghai Jiao Tong University Affiliated Sixth People’s Hospital, Shanghai, China; ^2^Department of Data and Analytics, WuXi Diagnostics Innovation Research Institute, Shanghai, China; ^3^PET Center, Huashan Hospital, Fudan University, Shanghai, China; ^4^State Key Laboratory of Brain and Cognitive Sciences, Institute of Biophysics, Chinese Academy of Sciences, Beijing, China

**Keywords:** Alzheimer’s disease, urinary formic acid, formaldehyde, biomarker, β-amyloid

## Abstract

**Introduction:**

The accumulation of endogenous formaldehyde is considered a pathogenic factor in Alzheimer’s disease (AD). The purpose of this study was to investigate the relationship between urinary formic acid and plasma biomarkers in AD.

**Materials and methods:**

Five hundred and seventy-four participants were divided into five groups according to their diagnosis: 71 with normal cognitive (NC), 101 with subjective cognitive decline (SCD), 131 with cognitive impairment without mild cognitive impairment (CINM), 158 with mild cognitive impairment (MCI), and 113 with AD.

**Results:**

With the progression of the disease, urinary formic acid levels showed an overall upward trend. Urinary formic acid was significantly correlated with Mini-Mental State Examination (MMSE) scores, the Chinese version of Addenbrooke’s Cognitive Examination III (ACE-III) scores, and Montreal Cognitive Assessment-Basic (MoCA-B) time. The areas under the receiver operating characteristic curves (AUC) of urinary formic acid in distinguishing NC from AD was 0.797, which was similar to that of plasma neurofilament light chain (NfL; AUC = 0.768) and better than other plasma biomarkers (Aβ40, Aβ42, Aβ42/Aβ40, T-tau, P-tau181, and P-tau181/T-tau). We also found that using urinary formic acid and formaldehyde levels could improve the accuracy of using plasma biomarkers to determine AD disease stage.

**Discussion:**

Our study revealed the possibility of urinary formic acid as a potential novel biomarker for the early diagnosis of AD.

## Introduction

Alzheimer’s disease (AD) is the most common form of dementia, characterized by progressive cognitive and behavioral disorders. The main pathological features of AD include abnormal accumulation of extracellular β-amyloid (Aβ), abnormal accumulation of neurofibrillary tangles of Tau protein, and synaptic damage (Wu et al., 2015). The pathogenesis of AD is not fully understood. What can be determined from current research is that AD is a continuous and concealed chronic disease, meaning AD can develop and last for many years before the emergence of evident cognitive impairment. The entire course of AD is divided into prodromal, preclinical, and dementia stages. Progression from subjective cognitive decline (SCD) to mild cognitive impairment (MCI) occurs before the irreversible dementia stage of AD, and this is the golden window for intervention and treatment (Khan et al., 2020).

Given the aging of the global population and the enormous social costs caused by AD, large-scale early screening of AD is necessary (Alzheimer’s Association, 2016). Sensitive neuropsychological measurement is complex and time-consuming, thus it is difficult to perform routinely for the elderly population. Positron emission tomography–computed tomography (PET-CT) scans can detect early Aβ deposits, but this technique is expensive and exposes patients to radiation. Biomarkers mainly come from invasive cerebrospinal fluid (CSF) and plasma tests, which seem to be effective for the early diagnosis of AD (Dubois et al., 2016; Jack et al., 2018; Graff-Radford et al., 2021). The composition of urine is complex and can reflect sensitive changes to metabolism and injury. Some studies have demonstrated that urinary biomarkers have the potential to screen for patients with AD (Tong et al., 2017).

In recent years, abnormal formaldehyde metabolism has been recognized as one of the essential features of age-related cognitive impairment (Yu et al., 2014; He, 2017). Our previous study reported a correlation between urinary formaldehyde levels and cognitive function, suggesting that urinary formaldehyde is a potential biomarker for the early diagnosis of AD (Wang et al., 2022). Formaldehyde plays a vital role in cellular metabolism and is involved in one-carbon metabolism, providing carbon for synthesizing and modifying biological compounds such as DNA, RNA, and amino acids (Li et al., 2021; Morellato et al., 2021). In the brain, formaldehyde can promote spatial memory formation under physiological conditions, and high formaldehyde concentrations can lead to protein denaturation and impair memory function (Tulpule and Dringen, 2013; He, 2017; Kou et al., 2022). Some studies have reported that formaldehyde concentrations were higher in the brains of patients with AD (He et al., 2010). It is well known that the aggregation of Aβ is an essential pathological mechanism and a characteristic of AD (Huang et al., 2021). Formaldehyde has been found to cross-link non-toxic Aβ monomers to form toxic dimers or oligomers (Fei et al., 2021; Zhao et al., 2021). Aβ can also induce formaldehyde production (Fei et al., 2021). Formic acid is a metabolic product of formaldehyde, and some formic acid is excreted in the urine in the form of formate (Morrow et al., 2015). Urinary formic acid reflects the metabolism of formaldehyde and has the potential to be a biomarker for the diagnosis of clinical housekeeping progression in AD.

We aimed to explore the relationship between urinary formic acid levels and cognitive changes throughout the progression of AD. We further analyzed the relationship between urinary formic acid and the apolipoprotein E (*APOE*) allele ε4, a high-risk gene for AD (Serrano-Pozo et al., 2021). The *APOE* ε4 allele is the most important genetic risk factor for AD after age 65, and it is associated with various pathological changes and cognitive impairment in AD. We compared the diagnostic effects of several plasma biomarkers and urinary formic acid, as well as the effects of Aβ precipitation on urinary formic acid. Finally, we analyzed the level of urinary formaldehyde to see if there were synergistic effects or differences between the two urinary indicators in diagnosis. Our systematic evaluation revealed that urinary formic acid could be a novel biomarker for early diagnosis of AD.

## Materials and methods

### Participants

The study included 574 participants recruited from the Memory Clinic of Shanghai Sixth People’s Hospital, China, and via advertising. All participants underwent cognitive function tests by trained staff in a neuropsychology assessment room between November 2019 and June 2021. According to the clinical diagnosis obtained from the cognitive function tests, the participants were divided into five groups: 71 normal cognitive (NC), 101 SCD, 131 cognitive impairment without mild cognitive impairment (CINM), 158 MCI, and 113 AD. The diagnosis of SCD adopted the standards of Jessen and other researchers (Jessen et al., 2014; Huang et al., 2018; Miebach et al., 2019; Jessen et al., 2020). CINM exhibited a single cognitive symptom and objective subtle cognitive decline. The criteria for MCI referred to the actuarial neuropsychological method proposed by Thomas et al. (2018) and Bondi et al. (2014). The diagnosis of AD was based on the National Institute on Aging–Alzheimer’s Association (NIA-AA) criteria (McKhann et al., 2011). Written informed consent was obtained from all participants or their caregivers. The ethics committee of Shanghai Jiao Tong University Affiliated Sixth People’s Hospital approved this study.

### Clinical assessments

All participants took standardized neuropsychological tests, including the Mini-Mental State Examination (MMSE) (Katzman et al., 1988), Montreal Cognitive Assessment-Basic (MoCA-B) (Huang et al., 2018), the Chinese version of Addenbrooke’s Cognitive Examination III (ACE-III) (Pan et al., 2022), and the Shape Trial Test (SST), Auditory Verbal Learning Test (AVLT) (Considine et al., 2017), Animal Fluency Test (AFT), and other tests which involved memory, language, attention, executive function, and visuospatial ability. Well-trained assessors conducted neuropsychological tests on all participants in Mandarin and recorded detailed clinical data.

#### Analysis of urine formaldehyde by high-pressure liquid chromatography

We collected morning urine from participants who did not abuse alcohol or drugs in the same week after the neuropsychological test. The urine sample was centrifuged at 4°C and 12,000 rpm for 10 min. The urine supernatant 0.4 ml was mixed with 2, 4-dinitrophenylhydrazine (DNPH, final concentration 0.1 g/L acetonitrile) and 0.1 ml trichloroacetic acid. The sample was rotated violently for 30 s, then centrifuged at 4°C, and 12,000 rpm for 10 min. The supernatant was transferred to a 2 ml glass bottle and heated in a water bath at 60°C for 30 min. A high-performance liquid chromatography system analyzed the supernatant with an ultraviolet detector (LC-20A, Shimadzu, Japan). The mobile phase was 65% acetonitrile-water, the flow rate was 0.8 ml/min, the column temperature was 35°C, the retention time was 6–7 min, and the detection wavelength was 355 nm. The high-pressure liquid chromatography (HPLC) traces of formaldehyde detection were shown in Supplementary Figure 1.

#### Measurement of urine formic acid

The levels of formic acid in collected urine samples were determined by the Formate Assay Kit (ab111748, Abcam, Cambridge, UK), following the protocol from the manufacturer. Ten microliter of urine was used per well, and the absorbance at 450 nm was measured on a 96-well microplate reader (SpectraMax Paradigm Multi-Mode, Molecular Devices, San Jose, CA, USA). The concentration of formic acid was calculated according to the standard curve.

### 18F-florbetapir positron emission tomography acquisition and analysis

One hundred and ninety-five participants were scanned by 18F-florbetapir PET (Biograph mCT Flow PET/CT; Siemens, Erlangen, Germany) at the PET Center of Huashan Hospital of Fudan University within 1 month of recruitment into the study. The subjects received an intravenous injection of 18F-AV-45 at a dose of about 10 mCi (370 MBq) and rested for 50 min. Then, PET imaging was performed for 20 min using low-dose CT. After the acquisition, the filtered back-projection algorithm reconstructed the PET image; attenuation, normalization, dead time, photon attenuation, scattering, and random coincidence were corrected. The results were determined independently by three clinicians who were blinded to the clinical diagnosis. Any differing opinions were resolved using the criterion that the global amyloid standardized uptake value ratio (SUVR, whole gray matter/bilateral cerebellar calf uptake value) was <1.29.

### Apolipoprotein E genotyping

Using a spin column DNA separation kit (Shanghai General Biotechnology Co., Ltd., Shanghai, China), genomic DNA was extracted from whole blood samples according to the manufacturer’s instructions. The two polymorphic sites of the *APOE* gene, rs.429358 and rs.7412, were identified by ligase detection reaction (LDR) using fluorescent nanospheres (New England Biolabs, Ipswich, MA, USA). Multi-ligase amplification was carried out using fluorescence-labeled magnetic nanospheres combined with upstream LDR probes and downstream labeled probes with unique fluorescent groups at each single-nucleotide polymorphism (SNP) site. The amplified LDR products were separated with the magnetic nanospheres and scanned for fluorescence spectra.

### Blood biomarker measurements

Blood samples were centrifuged, equally separated, and stored at −80°C until use. According to the manufacturer’s instructions (Chong et al., 2021), all biomarkers were measured on a single-molecule array (SIMOA) HD-1 analyzer platform (Quanterix, Billerica, MA, USA). The concentration of phosphorylated tau181 (P-tau181) in plasma was determined using an ultra-sensitive SIMOA immunoassay with AT270 mouse monoclonal antibodies against the threonine-181 phosphorylation site. Plasma Aβ40, Aβ42, and total tau (T-tau) were measured using a 3-plex A kit. The SIMOA NF-light VR Advantage kit detected neurofilament light chain (NfL; Quanterix, Billerica, MA, USA).

### Statistical analysis

One-way analysis of variance (ANOVA) was used to evaluate differences in age, education level, cognitive and neuropsychological test scores, and mean concentrations of urinary formic acid and urinary formaldehyde among the different diagnostic groups. Bonferroni multiple comparison tests were used in the post-test. Comparisons of continuous values between the *APOE* ε4+ and *APOE* ε4− groups and the Aβ+ and Aβ− groups were tested using the Mann–Whitney U test. If appropriate, χ^2^ or the Fisher exact test was used to test differences between different ratios. For all quantitative data, the results are expressed as mean ± standard deviation. Spearman’s rank correlation coefficient was used to evaluate the correlation between urinary formic acid and formaldehyde levels and cognitive scores or plasma biomarkers. Receiver operating characteristic (ROC) curves were used to assess the diagnostic strength of urinary formic acid, formaldehyde, and plasma biomarkers. *P*–values <0.05 were considered statistically significant. All analyses were carried out using SPSS software (v 22.0; IBM Corp., Armonk, NY, USA) and R software (v. 4.0.3; R Foundation, Vienna, Austria).

## Results

### Demographic and clinical characteristics of diagnostic groups

A total of 574 subjects were divided into 5 diagnostic groups, including NC (*n* = 71), SCD (*n* = 101), CINM (*n* = 131), MCI (*n* = 158), and AD (*n* = 113). Urinary formic acid and formaldehyde levels were measured in all patients. Table 1 shows detailed descriptive statistics of essential demographic and clinical characteristics of the five diagnostic groups. There were no significant differences in age, sex, or body mass index among the five groups. The neuropsychological test scores controlling the years of education in the AD group were significantly different from those in the NC group (*P* < 0.01). In the AD group, educational levels were low and cognitive impairment was severe (*P* < 0.05). We performed correlation analysis between urinary formaldehyde and formic acid levels and age; there were no significant differences in urinary formaldehyde and formic acid at different ages (Supplementary Figure 2).

**TABLE 1:** Demographics, disease characteristics, urinary formaldehyde and formic acid of five diagnostic groups.

	NC (*n* = 71)	SCD (*n* = 101)	CINM (*n* = 131)	MCI (*n* = 158)	AD (*n* = 113)	*P*-value (five groups)
Age (years)	64.0 ± 7.3	64.2 ± 5.8	65.6 ± 5.8	65.8 ± 6.4	65.8 ± 7.0	0.199^a^
Male (%)	24(33.8%)	27(26.7%)	42(32.1%)	49(31.0%)	44(38.9%)	0.420^b^
Education(years)	12.2 ± 2.9	12.1 ± 2.9	11.4 ± 2.8	10.9 ± 3.1	8.7 ± 4.0	0.000 ^a^
*APOE* ε4 + (%)	19(26.8%)	26(26.3%)	21(16.0%)	43(27.2%)	50(44.6%)	0.000^b^
BMI	24.4 ± 9.6	26.2 ± 18.0	24.2 ± 3.8	23.9 ± 2.8	23.7 ± 3.8	0.278^a^
MMSE	28.5 ± 1.4	28.1 ± 1.6	27.4 ± 1.7	26.6 ± 1.9	16.5 ± 5.2	0.000 ^a^
MoCA-B	26.5 ± 1.9	25.2 ± 3.8	23.8 ± 3.4	21.8 ± 3.4	11.2 ± 5.6	0.000^a^
ACE-III	84.2 ± 6.9	80.7 ± 7.6	76.7 ± 6.7	71.8 ± 8.2	45.4 ± 14.1	0.000^a^
BNT	25.3 ± 2.4	24.3 ± 4.3	23.4 ± 3.6	22.4 ± 3.4	19.1 ± 6.1	0.000^a^
AVLT-DR	6.5 ± 2.0	5.7 ± 2.2	3.4 ± 1.9	2.4 ± 2.2	0.5 ± 1.1	0.000^a^
AVLT-RC	22.3 ± 1.6	22.0 ± 1.6	21.1 ± 2.1	17.9 ± 2.8	14.5 ± 4.2	0.000^a^
AFT	17.6 ± 3.6	16.6 ± 3.8	14.4 ± 3.9	12.7 ± 3.5	8.8 ± 5.2	0.000^a^
STT-1(s)	43.6 ± 12.6	47.3 ± 13.3	49.9 ± 14.5	57.1 ± 21.5	71.6 ± 33.9	0.000^a^
STT-2(s)	112.5 ± 29.1	125.4 ± 41.2	134.3 ± 44.6	154.0 ± 46.8	176.1 ± 62.9	0.000^a^
Urinary formic acid	0.18 ± 0.07	0.26 ± 0.16	0.24 ± 0.12	0.26 ± 0.14	0.28 ± 0.14	0.000^a^
Urinary formaldehyde	8.54 ± 3.26	10.56 ± 4.65	9.10 ± 4.45	9.47 ± 4.99	10.81 ± 5.73	0.003^a^

*^a^*One way ANOVA.

^b^χ^2^ test.

BMI, body mass index; MMSE, mini-mental state exam; MoCA-B, montreal cognitive assessment-basic; ACE-III, the chinese version of addenbrooke’s cognitive examination III; AFT, animal fluency test; STT, shape trial test; AVLT-DR, auditory verbal learning test delay recall; AVLT-RC, auditory verbal learning test recognition. Data were shown as mean ± standard deviation.

### Relationship between urinary formaldehyde, urinary formic acid, and cognitive ability

**FIGURE 1:**
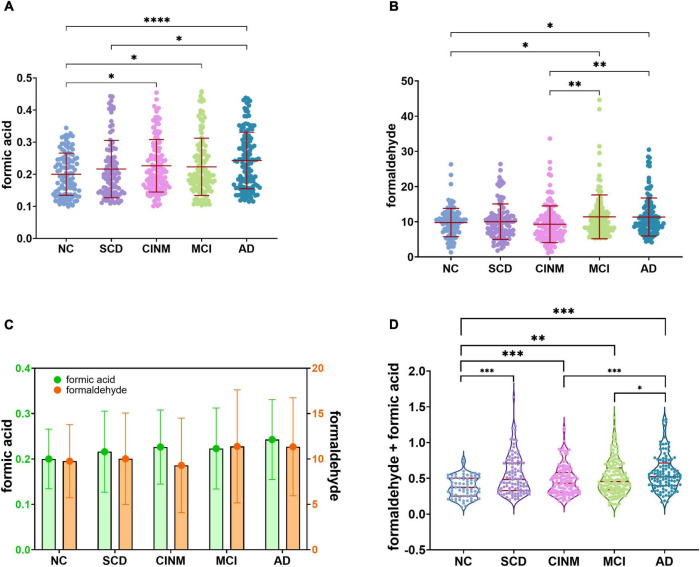
Urinary formaldehyde and formic acid level are associated with cognitive abilities of Alzheimer’s disease (AD). **(A)** With the progress of the disease, urinary formic acid showed an overall upward trend but decreased slightly in the mild cognitive impairment (MCI). **(B)** The level of urinary formaldehyde decreased in cognitive impairment without mild cognitive impairment (CINM) and reached its peak in MCI. **(C)** The trend of two urinary biomarkers shows a “double turning” points. **(D)** Normalized sum of formic acid and formaldehyde levels across various AD stages. Normalization formula: *z*_*i*_ = [*x*_*i*_ – min(x)]/[max(x) – min(x)]. *z*_*i*_, the *i*th normalized value in the dataset; *x*_*i*_, the *i*th value in the dataset. min(x), the minimum value in the dataset; max(x), the maximum value in the dataset. *p < 0.05; **p < 0.01; ***p < 0.001, and ****p < 0.0001.

Compared to the NC group, urinary formic acid levels were significantly higher in the SCD, CINM, MCI, and AD groups (all *P* < 0.05). Overall, urinary formic acid levels were slightly lower in the CINM group compared with the SCD, MCI, and AD groups, but there was no statistical difference between them. Urinary formaldehyde levels were significantly higher in AD than in NC (*P* < 0.05), but no statistical difference was observed between the remaining groups. The urinary formaldehyde levels showed a similar “up-down-up” trend as the urinary formic acid levels, and the urinary formic acid level was lower in CINM (Figure 1). As formic acid is a metabolic product of formaldehyde, we compared the levels of formic acid plus formaldehyde across different AD stages, and the results also showed that the levels of formic acid plus formaldehyde were significantly higher in SCD, CINM, MCI, and AD compared to NC. Moreover, we found that the levels of formic acid plus formaldehyde were significantly higher in AD than in CINM and MCI (Figure 1D).

**FIGURE 2:**
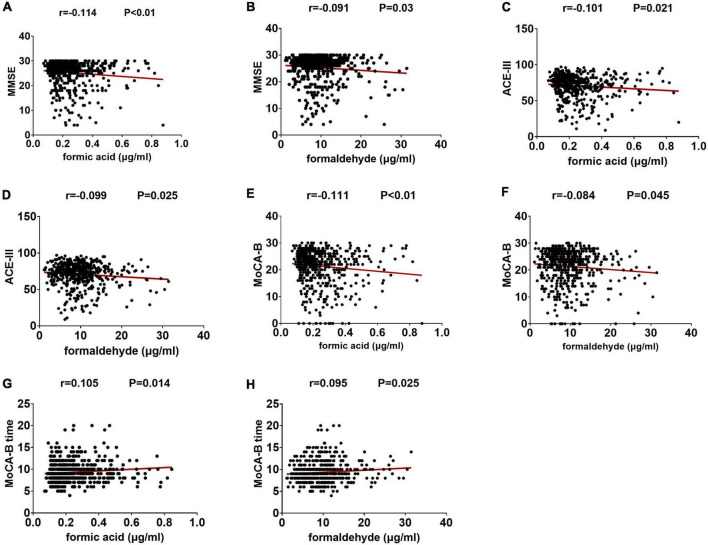
The levels of two kinds of urine markers are related to the neuropsychological test score. **(A)** Correlation between formic acid and MMSE. **(B)** Correlation between formaldehyde and MMSE. **(C)** Correlation between formic acid and ACE-III. **(D)** Correlation between formaldehyde and ACE-III. **(E)** Correlation between formic acid and MoCA-B. **(F)** Correlation between formaldehyde and MoCA-B. **(G)** Correlation between formic acid and MoCA-B time. **(H)** Correlation between formaldehyde and MoCA-B time.

We estimated the relationship between these two urinary biomarkers and cognitive ability levels. Urinary formaldehyde levels were negatively correlated with MMSE scores (*r* = −0.091, *P* < 0.05), ACE-III scores (*r* = −0.099, *P* < 0.05), and MoCA-B scores (*r* = −0.084, *P* < 0.05), and they were positively correlated with MoCA-B time (*r* = 0.095, *P* < 0.05). Similar, slightly higher correlations were seen between urinary formic acid and MMSE scores (*r* = −0.114, *P* < 0.01), ACE-III scores (*r* = −0.101, *P* < 0.05), MoCA-B scores (*r* = −0.111, *P* < 0.01), and MoCA time (*r* = 0.105, *P* < 0.05) (Figure 2).

The two urine biomarkers showed different correlations with cognitive domain scores of the ACE-III (Table 2). Urinary formic acid showed a negative correlation with attention and memory scores (*P* < 0.05), while visuospatial and memory scores correlated with urinary formaldehyde levels (*P* < 0.05).

**TABLE 2:** Correlation of urinary formic acid and urinary formaldehyde for each cognitive domain score in the Chinese version of Addenbrooke’s cognitive examination III (ACE-III).

		Attention	Memory	Verbal fluency	Language	Visuospatial skill
Urinary formic acid	R	−0.133*	−0.107*	–0.044	–0.061	–0.070
	*P* value	0.002	0.015	0.317	0.167	0.112
Urinary formaldehyde	*r*	–0.076	−0.104*	–0.084	–0.053	−0.106*
	*P* value	0.086	0.019	0.056	0.234	0.016

*P-value <0.05.

### Effect of brain Aβ accumulation on urinary formic acid and formaldehyde in different diagnostic groups

**FIGURE 3:**
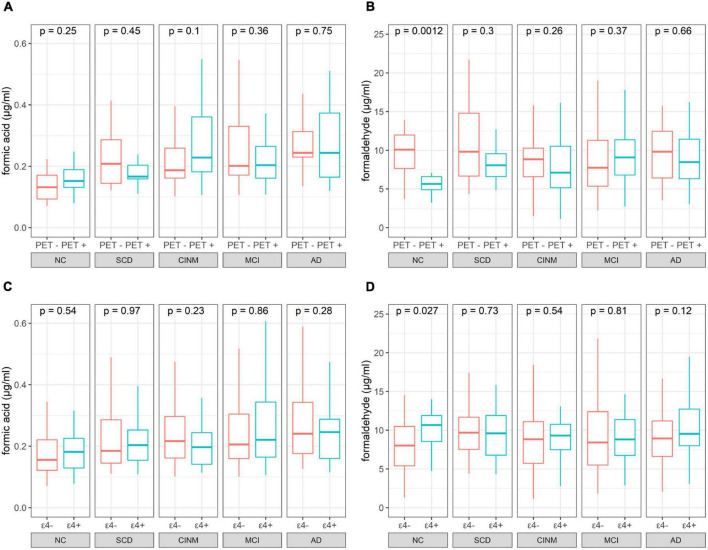
Boxplot of urinary indicator levels separated by PET status and apolipoprotein E (*APOE*) ε4 status. **(A)** Formic acid levers of PET negative and positive across normal cognitive (NC), subjective cognitive decline (SCD), cognitive impairment without mild cognitive impairment (CINM), mild cognitive impairment (MCI), and Alzheimer’s disease (AD) subgroup. **(B)** Formaldehyde levers of PET negative and positive across NC, SCD, CINM, MCI, and AD subgroup. **(C)** Formic acid levels of *APOE* ε4 negative and positive across NC, SCD, CINM, MCI, and AD subgroup. **(D)** Formaldehyde levels of *APOE* ε4 negative and positive across NC, SCD, CINM, MCI, and AD subgroup. The *P*-values were obtained by Mann–Whitney U test.

Two hundred and forty-five participants underwent 18F-florbetapir PET imaging to identify Aβ deposits. We further analyzed the difference between urinary formic acid and formaldehyde in different amyloid states. Based on plaque presence, we divided the five diagnostic groups into Aβ− and Aβ+ subgroups. The Aβ− group included 13 cases in the NC group, 30 SCD cases, 33 CINM cases, 45 MCI cases, and 11 AD cases. The Aβ+ group had 12 NC cases, 13 SCD cases, 24 CINM cases, 24 MCI cases, and 40 AD cases. Urinary formic acid levels were not significantly different between the subgroups in the NC, SCD, CINM, MCI, and AD diagnostic groups (Figure 3A). Urinary formaldehyde levels in the Aβ− subgroup were higher than that in the Aβ+ subgroup in patients in the NC group (*P* = 0.0012), while there were no significant differences in the other diagnostic groups (Figure 3B).

### Effect of apolipoprotein E genotype on urinary formic acid and formaldehyde in different diagnostic groups

We evaluated the relationship between the *APOE* ε4 alleles on urinary formic acid and formaldehyde concentrations in each diagnostic group. *APOE* genotyping was performed in all participants. According to the presence or absence of *APOE* allele ε4, the subjects in each diagnostic group were divided into *APOE* ε4+ (ε2/ε4, ε3/ε4, ε4/ε4) and *APOE* ε4− (ε2/ε2, ε2/ε3, ε3/ε3) groups. Shown based on diagnostic group, this resulted in: NC, 52 *APOE* ε4− and 19 *APOE* ε4+; CINM, 73 *APOE* ε4− and 26 *APOE* ε4+ in CINM; MCI, 115 *APOE* ε4− and 43 *APOE* ε4+; and AD, 62 *APOE* ε4− and 50 *APOE* ε4+. Urinary formic acid levels were not significantly different in NC, SCD, CINM, MCI, and AD between the subgroups (Figure 3C). Urinary formaldehyde levels were higher in the *APOE* ε4+ subgroup than the *APOE* ε4− subgroup in NC (*P* = 0.027), while there were no significant differences in the other diagnostic groups (Figure 3D). As formic acid is a metabolic product of formaldehyde, we analyzed formic acid plus formaldehyde to differentiate between *APOE* ε4 genotypes and Aβ plaque states across various AD stages, and no significant differences were found (Supplementary Figure 3).

### Correlation analysis of Alzheimer’s disease plasma biomarkers and urinary formaldehyde and formic acid levels

To investigate the potential association between plasma markers and urinary formic acid and formaldehyde, we analyzed their correlation in 326 participants (Table 3). Urinary formaldehyde was positively correlated with T-tau (*r* = 0.110, *P* < 0.05) and negatively correlated with Aβ42 (*r* = −0.162, *P* < 0.01), the Aβ42/Aβ40 ratio (*r* = −0.180, *P* < 0.01) and the P-tau181/T-tau ratio (*r* = −0.128, *P* < 0.05). There was no correlation between urinary formic acid levels and these plasma biomarkers.

**TABLE 3:** Correlation analysis of Alzheimer’s disease (AD) plasma biomarkers and urinary biomarkers.

		T-tau	P-tau181	P-tau181/Ttau	Aβ 42	Aβ 40	Aβ 42/Aβ 40	NfL
Urinary formic acid	*r*	0.041	0.086	0.002	0.044	0.006	0.065	–0.005
	*P* value	0.461	0.121	0.973	0.426	0.920	0.239	0.929
Urinary formaldehyde	*r*	0.110*	–0.061	−0.128*	−0.162*	0.30	−0.180*	–0.072
	*P* value	0.047	0.276	0.021	0.003	0.590	0.001	0.195

NfL, neurofilament light chain. *P-value <0.05.

**FIGURE 4:**
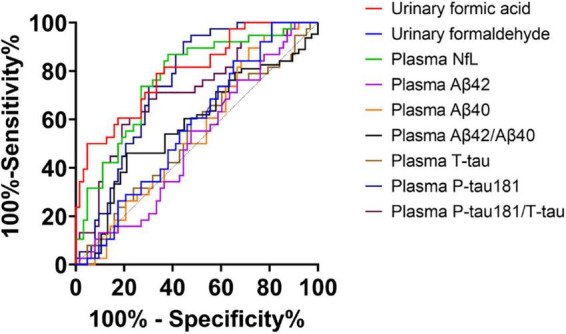
Receiver operating characteristic (ROC) curve of two urine biomarkers and plasma biomarkers for diagnosis of normal cognitive (NC) and Alzheimer’s disease (AD). Urinary formic acid: AUC = 0.797, *P* < 0.001, standard >0.2119, sensitivity: 66.7%, specificity: 78.9%; Urinary formaldehyde: AUC = 0.571, *P* > 0.05; Plasma neurofilament light chain (NfL): AUC = 0.768, *P* < 0.001, standard >18.506, sensitivity: 61.9%, specificity: 86.6%; Plasma T-tau: AUC = 0.537, P > 0.05; Plasma P-tau181: AUC = 0.749, *P* < 0.001, standard ≤3.037, sensitivity: 92.1%, specificity: 55.6%; Plasma P-tau181/T-tau: AUC = 0.711, *P* < 0.001, standard ≤0.6577, sensitivity: 57.9%, specificity: 81%; Plasma Aβ42: AUC = 0.506, *P* > 0.05; Plasma Aβ40: AUC = 0.544, *P* > 0.05; Plasma Aβ42/Aβ40: AUC = 0.575, *P* > 0.05.

Considering the application prospect of urine biomarkers as screening for cognitive impairment, we used ROC curves to analyze the discrimination level of plasma biomarkers and these two urine biomarkers in diagnosing NC and AD. NfL (AUC = 0.768, *P* < 0.001, standard >18.506, sensitivity: 61.9%, specificity: 86.6%), *P*-tau181 (AUC = 0.749, *P* < 0.001, standard ≤3.037, sensitivity: 92.1%, specificity: 55.6%) and *P*-tau181/*T*-tau (AUC = 0.711, *P* < 0.001, standard ≤0.6577, sensitivity: 57.9%, specificity: 81%) showed the best diagnostic effects among the plasma biomarkers, while the areas under the receiver operating characteristic curves (AUC) of urinary formic acid was superior to these three. Urine formaldehyde levels did not show good diagnostic value (Figure 4).

### Urinary biomarkers improved the prediction accuracy of plasma biomarkers for disease stage

**FIGURE 5:**
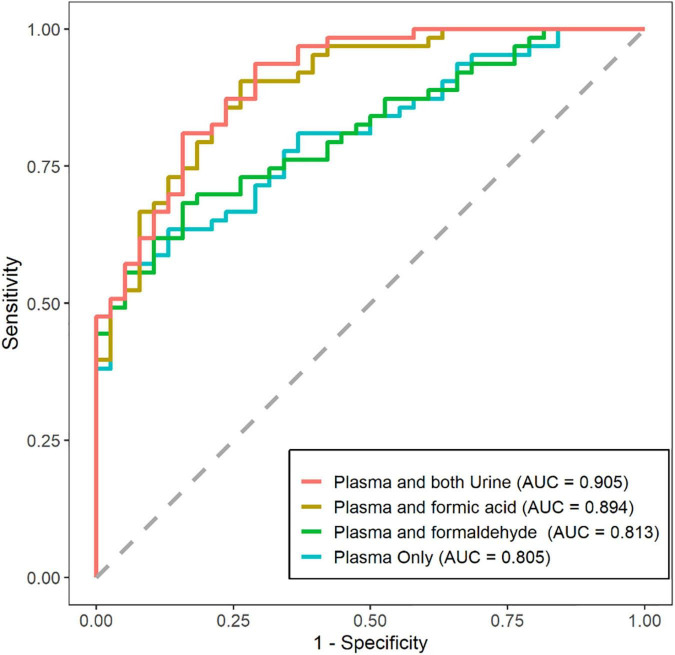
Prediction of disease stages by plasma and urine biomarkers alone versus plasma biomarkers only. Receiver operating characteristic (ROC) plots for showing the efficiency of logistic model for distinguishing the Alzheimer’s disease (AD) patients from normal cognitive (NC) patients. (NC: *n* = 38; AD: *n* = 63).

Studies have revealed that plasma biomarkers can predict disease stages, and integrating multiple plasma biomarkers could improve prediction accuracy. We investigated the ability to predict disease stages integrating plasma and urine biomarkers versus using only plasma biomarkers for the first time (Figure 5). The AUC was 0.805 (95% CI: 0.722, 0.888) using plasma biomarkers alone including Aβ40, Aβ42, T-tau, P-tau181, and NfL, and the AUC was 0.905 (95% CI: 0.847, 0.963) using the combined biomarkers. The AUC was 0.813 (95% CI: 0.733, 0.894) using the 5 plasma biomarkers and urinary formaldehyde, and the AUC was 0.894 (95% CI: 0.833, 0.956) using the 5 plasma biomarkers and urinary formic acid. These results suggested that urinary formic acid and formaldehyde could improve the prediction accuracy of plasma biomarkers for the disease stages of AD, and urinary formic acid levels showed more benefit than formaldehyde.

## Discussion

The development of AD occurs through long-term processes and is largely hidden. It is not possible to reverse neuronal damage and cognitive impairment even if the pathogenic factors, such as Aβ plaque accumulation, are cleared. Therefore, early diagnosis in the preclinical stage is crucial in the treatment of AD. Currently, the best biomarkers used to detect early-stage AD are the pathological biomarkers Aβ and Tau, detected using PET-CT scanning or CSF detection (Lista et al., 2014; Jack et al., 2018). However, PET-CT is expensive, and CSF examination is invasive. Plasma biomarkers, including Tau, NfL, and Aβ, are increasingly been used to diagnose and stage AD (De Wolf et al., 2020). Compared with invasive CSF and blood tests, urine testing is more suitable for large-scale screening (Tong et al., 2017). In the present study, we had four main findings. First, urinary formic acid levels were higher in the SCD, CINM, MCI, and AD diagnostic groups than in the NC group, and urinary formaldehyde levels were significantly higher in AD than in NC. Second, both urinary formic acid and formaldehyde levels were significantly negatively correlated with MMSE scores, ACE-III scores, and MoCA-B scores and positively correlated with MoCA-B time. Third, in the NC diagnostic group, urinary formaldehyde levels were higher in the *APOE* ε4+ subgroup than the *APOE* ε4− subgroup, and urinary formaldehyde levels were higher in the Aβ− subgroup than the Aβ+ subgroup. Fourth, urinary formic acid and formaldehyde levels could not only be used to differentiate between AD and NC, but they could improve the prediction accuracy for disease stage when combined with plasma biomarkers.

Research on urinary AD biomarkers has progressed, and some urinary biomarkers can be used to diagnose MCI or AD. However, there are no reports on urinary formic acid for the early diagnosis of AD (Praticò et al., 2002; Youn et al., 2011; Ma et al., 2015; García-Blanco et al., 2018). Studies have shown that excessive formaldehyde can cause “formaldehyde stress” and damage neurons (He et al., 2010; Zhao et al., 2021; Kou et al., 2022). In addition, formaldehyde can also initiate significant pathological changes related to AD, such as tau phosphorylation, tau aggregation, and Aβ deposition. Formaldehyde is considered a “trigger” of AD, meaning that capturing dynamic changes in formaldehyde can greatly help diagnose early AD (Tong et al., 2017). Our previous study explored the possibility of urinary formaldehyde as a potential biomarker for the early diagnosis of AD and obtained some positive results (Wang et al., 2022). Formic acid is a metabolic product of formaldehyde and can be excreted in the form of formate (Bruckner et al., 2008; Kageyama et al., 2008; Burgos-Barragan et al., 2017; Liu et al., 2017). Urinary formic acid can more sensitively reflect the metabolic changes of formaldehyde and is an important biomarker that has been neglected. In this study, we reported for the first time that urinary formic acid levels changed with the deterioration of cognitive function. Urinary formic acid showed a unique efficacy in the diagnosis of AD. In addition, there was a significant increase in urinary formic acid in the SCD diagnostic group, meaning that urinary formic acid can be used for the early diagnosis of AD. The present study once again showed that, with disease progression, urinary formaldehyde showed an “up-down-up” trend; urinary formaldehyde levels were high in SCD, significantly lower in CINM, and higher again in MCI and AD. Urinary formic acid levels were slightly lower in CINM, but this was still significantly higher than in NC. This suggests that may be a problem with clearance systems of the central nervous system in the early stages of AD.

An essential mechanism of AD is the imbalance between the production and clearance of metabolites in the brain (Querfurth and LaFerla, 2010; Gallina et al., 2015). Without a regular lymphatic system, the brain has a “glymphatic system” instead. The glymphatic system is based on healthy astrocytes, which excrete metabolic wastes from the brain, including Aβ and tau, to ensure the stability of the environment, which is crucial for neuron health (Guenette, 2003; Reeves et al., 2020). It was found that the aggregation of Aβ causes depolarization of aquaporin-4 (AQP4) on astrocytes. Moreover, the depolarization of AQP4 causes astrocytes to lose their excretory function, further aggravating the aggregation of metabolites such as Aβ (Lan et al., 2016). Astrocytes play an essential role in formaldehyde metabolism. Moreover, formaldehyde can promote the deposition of Aβ, thereby disrupting astrocyte function (Tulpule and Dringen, 2012). Impaired astrocytes will seriously affect the excretion of formaldehyde, resulting in a decrease of urine formaldehyde. In contrast, formic acid is more readily metabolized than formaldehyde, so urinary formic acid levels can remain high. No studies have reported the effects of the glymphatic system on formaldehyde and formic acid levels, which should be performed in the future.

The present study reported some noteworthy findings. There was a relationship between urinary formaldehyde levels, Aβ deposition, and the *APOE* ε4 allele. In brief, urinary formaldehyde levels were higher in the Aβ− and *APOE* ε4+ subgroups of the NC group. In addition, urinary formic acid and formaldehyde levels did not reflect the exact cognitive domains.

Many previous studies have shown that plasma biomarkers could predict disease stage and that integrating multiple plasma biomarkers could improve prediction accuracy (Palmqvist et al., 2021; West et al., 2021; Cheng et al., 2022). In the present study, we investigated the prediction ability for disease stages of integrating plasma and urine biomarkers compared to using plasma biomarkers alone, revealing that combining all biomarkers could improve prediction accuracy. Urinary formic acid levels showed higher usefulness than formaldehyde. Therefore, our study proved that the non-invasive and cost-effective urinary biomarkers improved the prediction accuracy of disease stages by plasma biomarkers. In view of some previous studies reported that the elevated expression of formaldehyde levels in diabetic rats, in cancer tissue, Parkinson’s disease, heart disease, and chronic liver disease (Tong et al., 2011; Rana et al., 2021; Zhang et al., 2021). The present study also showed that urine formaldehyde levels did not show a good diagnostic effect. Therefore, we believe that urine formaldehyde alone is not a useful biomarker for AD diagnosis, but it can be used as evidence for the cognitive deficit.

Admittedly, there are some limitations to the current research. This cross-sectional study cannot demonstrate causality, and the conclusions need to be verified by a long-term follow-up study. The explanations based on formaldehyde and formic acid metabolism remain theoretical, and corresponding animal experiments are needed to confirm this. The sample size for PET-CT data was not enough to analyze and draw reliable conclusions, especially in the NC diagnostic group. As PET-CT exposes patients to radiation and contrast medium must be used, therefore, most NC patients can’t accept to using PET-CT. We will increase the sample size to further prove the related conclusions in the future.

Urinary formic acid and formaldehyde are likely to be new biomarkers independent of the existing AD diagnostic criteria. We believe that further research can determine the best diagnostic models using urinary formic acid and formaldehyde levels to significantly improve the diagnostic efficiency of urine biomarkers in AD. Urine testing has unique advantages in early screening in the community. Using these urine biomarkers can significantly promote the popularity of early screening for AD, which can improve advice on diagnosis, treatment, and lifestyle for people at risk for AD. In-depth research on these biomarkers will also help to explore the mechanisms and potential treatments of AD. Dynamic changes in urinary formaldehyde and urinary formic acid suggest another new metabolic disorder in AD pathogenesis.

In conclusion, urinary formic acid levels changed dynamically related to the deterioration of cognitive function. Urinary formaldehyde levels were related to *APOE* ε4 genotype and the presence of Aβ depositions in the brain. Urinary formic acid and formaldehyde levels could not only be used for differentiation between AD and NC, but also could improve the prediction accuracy of plasma biomarkers for disease stages of AD. Our systematic evaluation revealed the novel possibility of urinary formic acid as a potential biomarker for the early diagnosis of AD.

## Data availability statement

The raw data supporting the conclusions of this article will be made available by the authors, without undue reservation.

## Ethics statement

The studies involving human participants were reviewed and approved by the Ethics Committee of Shanghai Jiao Tong University Affiliated Sixth People’s Hospital. The patients/participants provided their written informed consent to participate in this study. Written informed consent was obtained from the individual(s) for the publication of any potentially identifiable images or data included in this article.

## Author contributions

ZF and QG: conceptualization. YG, FX, RH, XC, and JD: formal analysis and visualization. YfW, YiW, JZ, ZF, YaW, and QG: project administration. YfW, YiW, and JZ: writing. All authors read and approved the final manuscript.
