# Supplementary material for: Systematic evaluation of urinary formic acid as a new potential biomarker for Alzheimer’s disease
Source: Front Aging Neurosci. 2022 Nov 30;14:1046066. doi: 10.3389/fnagi.2022.1046066 (PMC9747776; doi:10.3389/fnagi.2022.1046066)
Supplement: Supplementary file 1 [file Image_1.PDF]

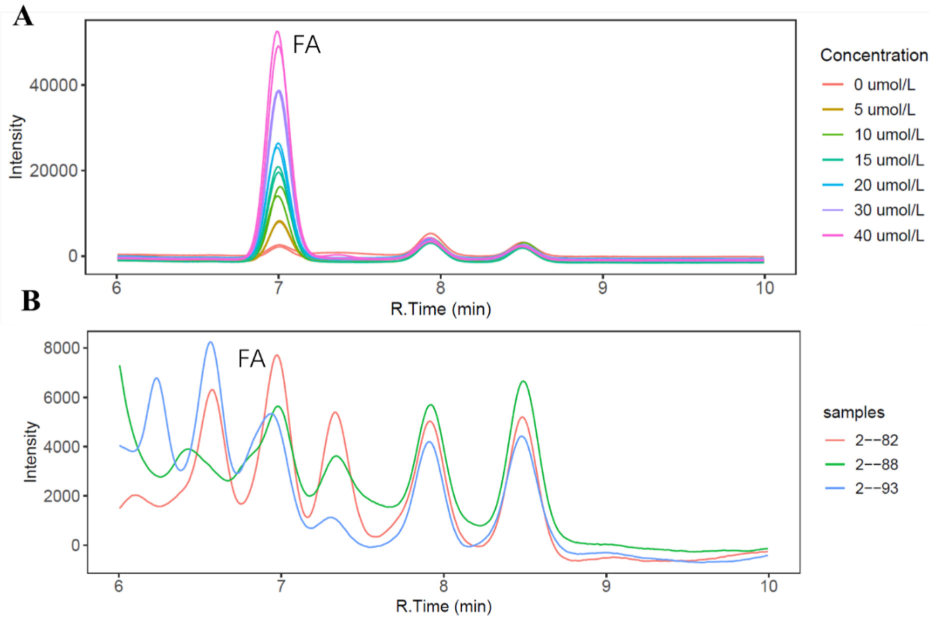

Supplementary Figure 1. The HPLC traces of formaldehyde detection. A. The representative HPLC curves of different concentrations of pure formaldehyde (0, 5, 10, 15, 20, 30, and 40 μmol/L). B. The representative HPLC curves of urine samples from 3 patients, respectively. FA: Formaldehyde.

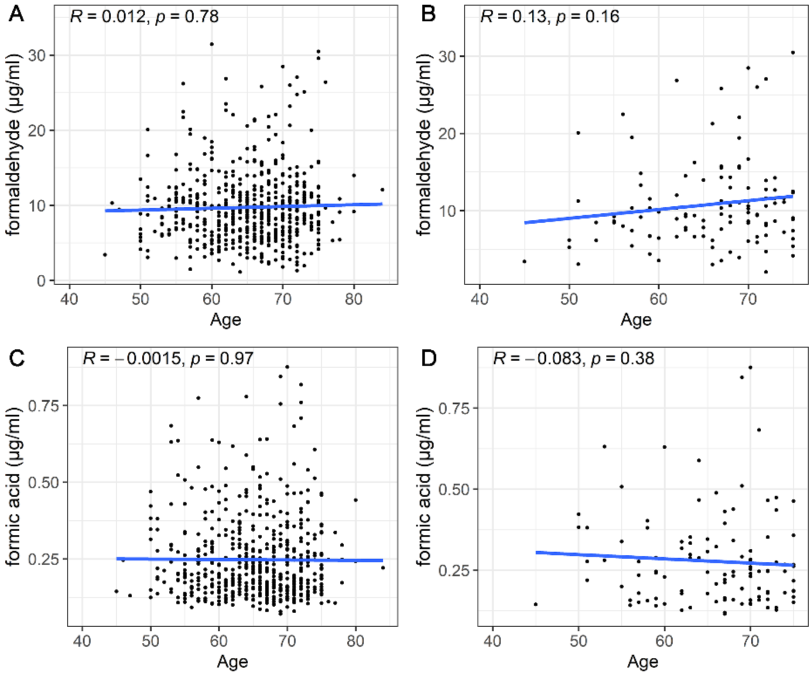

Supplementary Figure 2. Scatter plot of urinary biomarker levels and their association with age. A. Relationship between urinary formaldehyde and age in all 574 subjects. **B.** Relationship between urinary formaldehyde and age in 113 AD patients. **C.** Relationship between urinary formic acid and age in all 574 subjects. **D.** Relationship

between urinary formic acid and age in 113 AD patients.

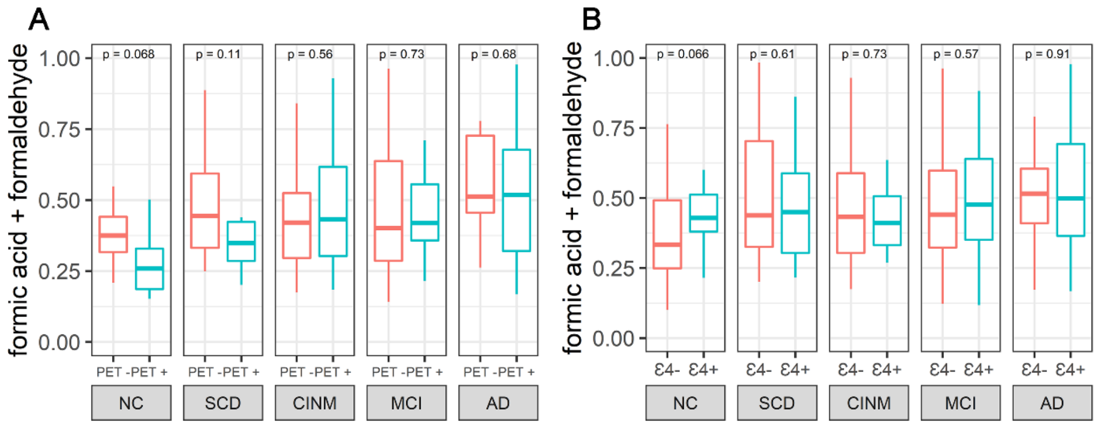

Supplementary Figure 3. Boxplot of urinary indicator levels separated by *APOE* ε4 genotype and Aβ deposition. A. Normalized sum of formaldehyde and formic acid levels stratified between PET status. **B.** Normalized sum of formaldehyde and formic acid levels stratified between *APOE* status. Normalization formula: $z_i = (x_i - \min(x)) / (\max(x) - \min(x))$. z_i: The ith normalized value in the dataset. x_i: The ith value in the dataset. min(x): The minimum value in the dataset. max(x): The maximum value in the dataset.
